# The Reach of Chronic-Disease Self-Management Education Programs to Rural Populations

**DOI:** 10.3389/fpubh.2014.00172

**Published:** 2015-04-27

**Authors:** Samuel D. Towne, Matthew Lee Smith, SangNam Ahn, Marcia G. Ory

**Affiliations:** ^1^Department of Health Promotion and Community Health Sciences, Texas A&M Health Science Center, School of Public Health, College Station, TX, USA; ^2^Department of Health Promotion and Behavior, College of Public Health, University of Georgia, Athens, GA, USA; ^3^Division of Health Systems Management and Policy, School of Public Health, The University of Memphis, Memphis, TN, USA

**Keywords:** chronic-disease self-management, evidence-based program, rural, intervention dose, older adults

## Abstract

This study assessed the sociodemographic characteristics of rural residents who participated in chronic-disease self-management education (CDSME) program workshops and the extent to which CDSME programs were utilized by those with limited access to health care services. We analyzed data from the first 100,000 adults who attended CDSME program workshops during a national dissemination spanning 45 states, the District of Columbia, and Puerto Rico. Approximately 24% of participants lived in rural areas. Overall, 42% of all participants were minorities; urban areas reached more minority participants (48%) than rural areas (25%). The average age of participants was high in rural (age, μ = 66.1) and urban (age, μ = 67.3) areas. In addition, the average number of chronic conditions was higher (*p* < 0.01) in rural (μ = 2.6 conditions) versus urban (μ = 2.4 conditions) areas. Successful completion of CDSME programs (i.e., attending four or more of the six workshop sessions) was higher (*p* < 0.01) in rural versus urban areas (78% versus 77%). Factors associated with higher likelihood of successful completion of CDSME programs included being Black (OR = 1.25) versus White and living in rural (versus urban) areas (OR = 1.09). Factors associated with lower likelihood of successful completion included being male (OR = 0.92) and residing in a primary care Health Professional Shortage Area or HPSA (versus a non-HPSA) (OR = 0.93). Findings highlight the capability of CDSME programs to reach rural residents, yet dissemination efforts can be further enhanced to ensure minorities and individuals in a HPSA utilize this program. Tailored strategies are needed to increase participant recruitment and retention in rural areas to overcome traditional barriers to health service access.

## Introduction

While it is known that individuals with chronic diseases are more likely to utilize health care services (1–3), we are still learning about their use of health promotion resources available in community settings. Further, less is known about the unique community characteristics and infrastructures that influence the delivery and adoption of evidence-based chronic-disease self-management education (CDSME) programs in traditionally underserved areas and populations.

Compared to metropolitan or urban areas, there is limited research about aging in rural areas. And, studies about rural populations are primarily demographic or epidemiological in focus. Disproportionately, more older adults live in rural areas (15% in rural, 12% in urban) (4), and rural areas have less health care service availability and fewer health care providers compared to urban areas (5–7). Relative to those living in urban areas, rural area residents are disproportionately affected by poor health outcomes and health care access barriers, which contributes to them having higher disease rates, disability rates, and risk factors for poor health outcomes (8–10).

Studies have shown that rural areas traditionally encounter geographic barriers limiting access to health care resources, as exemplified by areas designed as rural highly overlapping with health professional shortage area (HPSA) and medically underserved area designations (11, 12). Using geographic information systems (GIS), researchers have identified geospatial barriers hindering rural area residents, especially minority older adults, from accessing resources (e.g., longer distances, lower availability of health care providers) (13).

Prior research has documented the benefits of delivering evidence-based programs (EBP) in rural communities [e.g., improving health-related outcomes (14), falls efficacy (15)]. However, the extent to which CDSME programs are delivered in rural areas remains unknown. Because of the known effectiveness of CDSME programs (e.g., improved health outcomes, lower hospitalization, better chronic-disease management) (16–19), it is important to identify whether residents of rural areas have access to these EBP, especially in vulnerable rural areas with fewer health-related resources and services. Additionally, even when EBP are available in rural areas, it is important to assess whether or not participants in these areas attend enough sessions to receive adequate intervention dose. This is especially important considering individuals in rural areas may have greater distances to resources (e.g., health care resources), which may act as a barrier to program participation (8, 13).

As such, the objectives of this study were to: (1) assess the extent to which CDSME programs were utilized by rural residents and identify characteristics of these rural residents as compared to their urban counterparts; (2) investigate the geographic distribution of CDSME program participation based on the rurality of participants’ residence; and (3) examine factors associated with successful workshop completion.

## Materials and Methods

### Program description

With the goal of improving self-management skills among adults with chronic conditions, CDSME programs have been widely delivered across the US (20). The CDSME program suite of evidence-based self-management programs, developed at Stanford University Patient Education Research Center, uses the Social Learning Theory (21) to deliver these peer-led interventions (i.e., six sessions, once a week at 2.5 h each for six consecutive weeks) (20). The results of participation in this program include improved health, health care utilization (e.g., lower rate of hospitalizations) (19, 22), and health care cost savings (23).

### Data source and study population

We conducted a cross-sectional analysis using data collected via the national delivery (45 states, Puerto Rico, and the District of Columbia) (24) of the CDSME programs. As part of the American Recovery and Reinvestment Act of 2009, CDSME programs were delivered via the *Communities Putting Prevention to Work: Chronic-Disease Self-Management Program* initiative led by the US Administration on Aging in partnership with the Centers for Disease Control and Prevention (CDC) and the Centers for Medicare and Medicaid Services (CMS) (25). Analyses were conducted using data on the first 100,000 participants targeted in this initiative (25). Institutional Review Board approval for this study was given by Texas A&M University.

### Measures

#### Geospatial variables

Geospatial analyses were those examining differences across rurality. We were interested in characterizing participants and delivery sites by rural and urban categories. To accomplish this, the 2013 Area Health Resource File (AHRF) was used to identify geographic characteristics (i.e., rural residency, health professional resources) (26). We defined rurality based on county and separately ZCTA (ZIP Code Tabulation Areas)/ZIP Codes. For counties, urban influence codes (UIC) were merged with data from the National Council on Aging (NCOA) using Federal Information Processing Standards (FIPS) Codes. We compare results using both county and ZIP Code levels of rurality. We used county-level rurality in fully adjusted analyses. We dichotomized UIC into Metropolitan (UIC = 1–2) and Non-Metropolitan (UIC = 3–12) (27). For ZCTA/ZIP Codes, we merged Rural-Urban Commuting Area Codes (RUCA) into urban and non-urban (large rural cities, small rural towns, isolated small rural towns) areas (28). We also coded rurality into more than a two-way split (i.e., rural and urban). We coded rurality into a 4-way split including *Urban, Large Rural City/Town, Small Rural Town*, and *Isolated Small Rural Town*. These multiple rurality measurements allowed us to identify differences within rural areas with a greater degree of specificity in selected analysis.

Primary Care HPSA are defined based on geographic area, population groups, and facilities, with more detailed definitions available from the Health Resources Services Administration (HRSA) (http://www.hrsa.gov/shortage/) (29). Primary Care HPSAs were defined as either full, partial, or non-HPSA at the county level. A full-HPSA is defined as an entire county designated as a HPSA versus partial-HPSA. A non-HPSA is a county not designated as a HPSA.

Areas served by CDSME were defined as unique ZCTA/ZIP codes where at least one participant was located. These were spread nationwide throughout 9,599 unique ZCTA/ZIP Codes.

#### Dependent variable

Our primary dependent variable was successful workshop completion. Participant’s attendance was recorded to determine if adequate intervention dose was received. As defined by the program developers, a participant has “successfully” completed the program if they attended four or more of the six offered workshop sessions (19, 22, 25, 30).

#### Sociodemographics

Personal characteristics of the participants included age, sex, and race/ethnicity. We used one variable for race and ethnicity with categories of non-Hispanic White, non-Hispanic Black or African American, non-Hispanic Native American or Alaskan Native, non-Hispanic Asian American, and “other” race/ethnicity category (including non-Hispanic Native Hawaiian or other Pacific Islander, those identified as “other,” and those identified as belonging to multiple race/ethnic groups), and Hispanic. We also included living arrangement to specify whether participants lived alone or lived with others.

The number of chronic conditions among participants was identified as having any one or more of the following chronic diseases: diabetes, heart disease, hypertension, lung disease, arthritis, cancer, or “other” (another chronic disease). We summed the number of chronic diseases into one variable and grouped it into the following categories: one condition, two conditions, three conditions, four conditions, and five or more conditions (due to small sample sizes with six chronic conditions).

#### Statistical analyses

We conducted analyses on the first 100,000 participants reached in this initiative who had observations with complete data on all variables of interest. Those with missing data for age (*n* = 12,447), sex (*n* = 8,826), race/ethnicity (*n* = 12,124), living arrangement (*n* = 1,605), number of chronic conditions (*n* = 1,539), and geographic identifiers (*n* = 12,314) were omitted. Some participants had more than one of these exclusionary characteristics. Therefore our final sample size was 82,044. Analyses on observations with missing information (e.g., missing rurality) were not conducted because our primary goal was to measure outcomes across study characteristics (e.g., rurality). We did not attempt to measure program success independent of study characteristics.

We used independent sample *t*-tests and Chi Square for bivariate comparisons. Logistic regression analyses were used to investigate factors associated with successful workshop completion. We used logistic regression to predict the dichotomous outcome of successful completion (versus not attending at least four of the six workshop sessions). Fully adjusted analyses (logistic regression) includes participant race/ethnicity, rurality (county-level), HPSA status, participant sex, living arrangement (living alone or not), participant age, and the number of chronic conditions. SAS version 9.4 was used for all analysis (31). ArcGIS version 10.2 was used for mapping (32).

## Results

**Figure 1:**
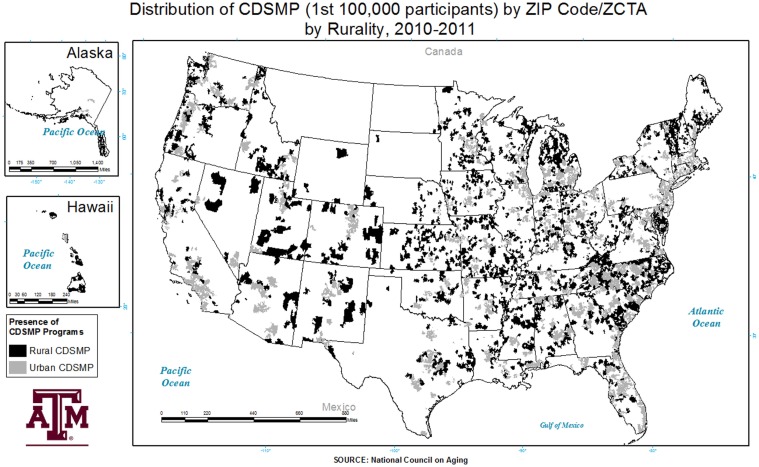
**Distribution of the chronic-disease self-management program by ZIP Code/ZCTA and rurality**.

Overall, 1,721 counties throughout the US had a CDSME program available to residents, while 1,421 counties did not offer a CDSME workshop. There were 922 rural counties and 799 urban counties offering CDSME workshops. Rural counties without a CDSME workshop totaled 1,130 versus 291 for urban counties. Here, 74.3% of areas lacking a CDSME workshop were rural. Some states had more workshop clustering, and others had wider coverage throughout the states (e.g., South Carolina and North Carolina). The distribution of rural CDSME program participants varied across the US (see Figure 1). Analysis across rurality indicated that approximately 22.1% (using county-level rurality) to 24.4% (using ZCTA/ZIP Code-level rurality) of CDSME program participants resided in rural areas.

Characteristics of participants across rurality are provided in Table 1. Age ranged from 18 to over a 100 across all observations. The bulk of participants were female (approximately 78.0%). Approximately 48.9% of participants lived alone. In general, participants had at least two chronic conditions, where the average number of chronic conditions was 2.5.

**Table 1:** **Distribution of key characteristics across rurality**.

	Rural		Urban		Total
	ZIP Code/ZCTA	County	ZIP Code/ZCTA	County	
Sample size	(*n* = 19,982) 24.38%*	(*n* = 18,111) 22.09%*	(*n* = 61,991) 75.62%*	(*n* = 63,862) 77.91%*	(*n* = 81,973) 100%
Number of chronic conditions	2.59*	2.59*	2.42	2.43	2.46
Sex (% Female)	77.70%	78.00%	78.06%	77.96%	77.97%
Age	66.06*	66.13*	67.32	67.27	67.01
Living alone	52.12%*	52.75%*	47.88%	47.83%	48.92%
Race/ethnicity
White	74.52%	75.48%	52.19%	52.57%	57.63%
Black	13.20%	13.21%	22.33%	22.06%	20.11%
AIAN	2.56%	2.48%	0.92%	0.99%	1.32%
Asian	1.07%	1.17%	3.99%	3.88%	3.28%
Other	6.77%	6.36%	13.11%	13.04%	11.56%
Hispanic	1.87%	1.29%	7.46%	7.46%	6.10%
HPSA
Full HPSA	8.48%	7.59%	34.12%	35.01%	42.60%
Partial HPSA	11.36%	10.26%	33.58%	34.68%	44.94%
Non-HPSA	4.52%	4.23%	7.94%	8.23%	12.46%

*Indicates significantly different (*p* < 0.01) from urban areas using independent group *t*-test for continuous variables (number of chronic conditions, percent female, age, and percent living alone). The overall sample size is different (*p* < 0.01) by rurality (Chi Square).

When compared by the geography of residence, participants residing in rural areas were younger (*p* < 0.01) on average compared to those in urban areas (approximately 66.1 years versus 67.3 years). The percent of individuals living alone was higher (*p* < 0.01) in rural areas (i.e., ranging from 52.1 to 52.6% in rural areas versus 47.9 to 47.8% in urban areas by ZCTA/ZIP Code and county, respectively). Participants residing in rural areas had more (*p* < 0.01) chronic conditions on average compared to those in urban areas (approximately 2.6 conditions versus 2.4 conditions).

Table 2 presents the successful completion rates by rurality. Successful completion of the CDSME program was uniformly high at 77.3% overall; however, it was slightly higher in rural areas (77.9%) than in urban areas (77.1%). When we specified a 4-level categorization for rurality, we found participants residing in large rural towns (78.4%) and isolated small rural towns (78.3%) had higher successful completion rates than those participants residing in small rural towns (76.6%).

**Table 2:** **Successful completion rates by rurality**.

	Successful completion	Standard deviation	Total (*n* = 82,044)
Rurality			
Urban	77.1%	0.42	62,051
Large rural city/town	78.4%	0.41	10,054
Small rural town	76.6%	0.42	5,900
Isolated small rural town	78.3%	0.41	4,039

*Operational definition of rurality (4-way) includes Urban: RUCA 1.0, 1.1, 2.0, 2.1, 4.1, 5.1, 7.1, 8.1, and 10.1; Large Rural City/Town: 3.0, 4.0, 4.2, 5.0, 5.2, 6.0, 6.1, 7.2, 8.2, and 10.2; Small Rural Town: 7.0, 7.3, 7.4, 8.0, 8.3, 8.4, 9.0, 9.1, 9.2, and 10.3; Isolated Small Rural Town: 10.0, 10.4, 10.5, and 10.6*.

Table 3 presents the distribution of areas with a CDSME program presence (i.e., having one or more CDSME workshops available in the county) by rurality. The majority of areas with CDSME workshops were urban (70.0%). Approximately 9.3% of all CDSMP workshops were located in isolated small rural towns, and approximately 8.2% were located in small rural towns. The average number of participants in areas with a CDSME workshop by rurality (calculated at the ZCTA/ZIP Code) was 9.2 participants in urban areas, which was almost twice the amount of participants in isolated small rural towns. Among areas with a CDSME workshop present, the range of the average number of participants in urban areas was much higher than small rural towns or isolated small rural towns (1–208 participants versus 1–62 participants and 1–45 participants, respectively). However, the highest range in the number of participants in a ZCTA/ZIP Code was measured in areas identified as a large rural city/town (1–884 participants).

**Table 3:** **Distribution of CDSMP sites (unique ZCTA/ZIP codes with a participant) by rurality**.

	Average number of participants	Standard deviation	Range		Total (*n* = 9,599)
Rurality				
Urban	9.23	13.67	1	208	6,725 (70.01%)
Large rural city/town	8.46	28.11	1	884	1,192 (12.42%)
Small rural town	7.40	9.37	1	62	791 (8.24%)
Isolated small rural town	4.54	5.50	1	45	891 (9.28%)

*Operational definition of rurality (4-way) includes Urban: RUCA 1.0, 1.1, 2.0, 2.1, 4.1, 5.1, 7.1, 8.1, and 10.1; large rural city/town: 3.0, 4.0, 4.2, 5.0, 5.2, 6.0, 6.1, 7.2, 8.2, and 10.2; Small Rural Town: 7.0, 7.3, 7.4, 8.0, 8.3, 8.4, 9.0, 9.1, 9.2, and 10.3; isolated small rural town: 10.0, 10.4, 10.5, and 10.6*.

Table 4 presents factors associated with successful completion of the CDSME program. A greater likelihood of successful completion was associated with being Black (OR = 1.25), or another race/ethnicity (OR = 1.32) versus being non-Hispanic White. A greater likelihood of successful completion was also associated with living in a rural county (OR = 1.10). Factors associated with lower likelihood of successful completion of the CDSME program included being male (OR = 0.92) and residing in a full-HPSA (OR = 0.93) versus a non-HPSA.

**Table 4:** **Likelihood of successful completion of the CDSMP**.

	Odds ratio	*p*-Value	Confidence intervals (95%)	
Race				
White (referent)				
Black	1.249*	<0.0001	1.194	1.305
AIAN	0.923	0.0023	0.804	1.060
Asian	1.209**	0.0342	1.098	1.331
Other	1.318*	<0.0001	1.246	1.395
Hispanic	0.994	0.0008	0.927	1.067
Rurality
Rural county	1.095*	<0.0001	1.051	1.140
HPSA
Non-HPSA (referent)				
Partial HPSA	0.988	0.1588	0.936	1.042
Full HPSA	0.926*	0.0002	0.877	0.977
Sex
Female (referent)				
Male	0.924*	<0.0001	0.888	0.961
Household status
Lives with others (referent)				
Lives alone	1.017	0.3376	0.983	1.052

*Indicates significant differences (*p* < 0.01) using logistic regression.

**Indicates significant differences (*p* < 0.05) using logistic regression.

## Discussion

Our findings support earlier work about rural–urban differences in access to health-related resources (33). As expected, CDSME programs were less prevalent in rural versus urban areas. However, this study highlights that CDSME workshops are reaching rural areas in the US, although this reach is less than 25% of all rural areas. This is critical because CDSME programs have been shown to facilitate improvements in health status and other health-related outcomes among adults. CDSME programs assist participants to set goals, problem solve and do action planning that can help in medical, emotional, and social role management of chronic conditions (16–18).

Rural residents face several issues related to health care and disease prevention program access (5–7). Identifying efficient ways to bridge access issues in rural areas is critically important for those who are older and have one or more chronic conditions. Improving the rural reach of EBP is one example of bridging this gap and linking rural residents to appropriate health care services intended to improve-health outcomes (34). Thus, examining strategies that bolster participation rates in rural and urban areas is warranted. More research is needed to identify why rural residents had somewhat higher completions rates when compared to urban residents. Overall, rural adults may be harder to reach and have other barriers related to social support, as exemplified by rural participants reporting higher rates of living alone (35, 36). In addition, the somewhat higher rates in the number of chronic conditions among rural residents may make this population potentially more vulnerable to self-care issues and in need of CDSME programs.

In the current study, the smaller number of participants in rural versus urban ZCTA/ZIP Codes may be related to the smaller number of eligible participants in these areas (i.e., population density and geographic isolation) (37) and the difficulty of some potential participants getting to centralized locations (e.g., longer distance, limited transportation) (38–41). To adequately serve rural populations, efforts are needed to ensure these programs are delivered in areas closer to potential/existing participants’ homes. Offering these programs in closer proximity to rural participants’ residences has potential to increase attendance rates because it can reduce the time and distance traveled to get to workshop sessions. Strategies make CDSME programs available rural residents’ homes include embedding programs into existing local community infrastructures such as health care clinics or agricultural extension health services. Engaging multiple delivery sites in these communities (through the aging services network and public health system) is encouraged. For example, offering programs in faith-based organizations have been shown to improve participant reach (34). Embedding these programs in as regular offerings in organizations where rural residents frequently attend may increase their participation and foster long-term program sustainability.

Another strategy to better serve rural communities with CDSME programs includes cross-training workshop facilitators to be certified to an array of EBP (e.g., disease self-management, fall prevention). Cross-training these facilitators can increase the capacity of rural areas to deliver a collection of diverse EBP, each of which differ in purpose to meet the needs of rural residents and their caregivers. While increasing the availability of EBP in rural communities is essential, increasing access (and repeated access) to workshops is of equal importance. Once recruited into the program, additional efforts are needed to ensure participants remain in the program long enough to receive sufficient intervention dose for desired effects. Possible strategies to improve participants’ access to and retention within workshops may include the creation of participant “buddy systems,” exploring options for free or low-cost transportation services (e.g., shared rides or volunteer drivers), including technologically driven approaches, or holding meetings in community settings where older adults are already congregating.

More research should be conducted to identify differences in how programs are delivered in rural versus urban areas (e.g., strategies for recruitment and retention of different community partners; targeting different delivery settings; and determining ideal but feasible class size). Further investigation is also needed to assess the health-related impact of programs in rural versus urban areas, with special attention to cost-benefit issues. Future efforts should also examine whether differences by region or US territory exist (e.g., comparisons between continental US and Hawaii/Puerto Rico).

### Limitations

The measure of rurality used in health services research is an important consideration in studies about rurality because the designated selection has potential to change areas of comparison and influence study findings (42). Our definition of rurality varied across the level of analysis. We used both a county-level measure (UIC) and the ZCTA/ZIP Code-level measure (RUCA), which assessed rurality in both larger areas (i.e., counties) and on a more micro-level (i.e., ZCTA/ZIP Codes). Thus, our use of different levels of rurality in this study provides a more complete picture of geospatial differences. While CDSME workshops were delivered in Puerto Rico, the measure of rurality used (i.e., 2006 RUCA Codes) was not available for Puerto Rico (43). As such, we were unable to provide accurate estimates of delivery by rurality for this area in the current study.

Data presented in the current study is based on the level of rural residents reached by the CDSME programs only among those who participated in this initiative. We do however, provide the rural reach by geographic distribution (i.e., reach within areas). Further, distances traveled by participants to attend workshops were not measured, thus we could not determine if time or distance traveled influenced workshop attendance. Additionally, the level of missing data is not uncommon to community-based interventions (44–46). While there was substantial missing data, this may have been attributed more to the sites’ administrative ability to collect field data than from individual data refusal (47). Because the analyses performed in this study were not longitudinal, we could not measure changes in the rural reach of the CDSME programs over time. Designing such longitudinal analyses is highly recommended as a next step in identifying whether progress is being made in reaching rural residents. We acknowledge that because of our large sample size seemingly small comparative differences were statistically significant. To be more conservative and protect against Type I error, we used a *p*-value of 0.01 in all study analyses.

## Conclusion

The current study helps lessen the gap in what is known about the rural reach of CDSME programs and factors affecting successful completion. Findings highlight the capability of CDSME programs to reach rural residents, yet dissemination efforts can be enhanced to ensure minorities and individuals in HPSAs utilize this program. Tailored strategies are needed to increase participant recruitment and retention in rural areas to overcome traditional barriers to health service access. Assessing the infrastructure in rural areas may be helpful for identifying viable partners for those seeking to deliver EBP to residents of rural areas, creating greater uptake, reach, and sustainability.

## Conflict of Interest Statement

The authors declare that the research was conducted in the absence of any commercial or financial relationships that could be construed as a potential conflict of interest.

This paper is included in the Research Topic, “Evidence-Based Programming for Older Adults.” This Research Topic received partial funding from multiple government and private organizations/agencies; however, the views, findings, and conclusions in these articles are those of the authors and do not necessarily represent the official position of these organizations/agencies. All papers published in the Research Topic received peer review from members of the Frontiers in Public Health (Public Health Education and Promotion section) panel of Review Editors. Because this Research Topic represents work closely associated with a nationwide evidence-based movement in the US, many of the authors and/or Review Editors may have worked together previously in some fashion. Review Editors were purposively selected based on their expertise with evaluation and/or evidence-based programming for older adults. Review Editors were independent of named authors on any given article published in this volume.
